# Translational Medicine in Uremic Vascular Calcification: Scavenging ROS Attenuates p-Cresyl Sulfate-Activated Caspase-1, NLRP3 Inflammasome and Eicosanoid Inflammation in Human Arterial Smooth Muscle Cells

**DOI:** 10.3390/life12050769

**Published:** 2022-05-23

**Authors:** Jia-Feng Chang, Hsiao-Ling Kuo, Shih-Hao Liu, Chih-Yu Hsieh, Chih-Ping Hsu, Kuo-Chin Hung, Ting-Ming Wang, Chang-Chin Wu, Kuo-Cheng Lu, Wei-Ning Lin, Chi-Feng Hung, Wen-Chin Ko

**Affiliations:** 1 Division of Nephrology, Department of Internal Medicine, En Chu Kong Hospital, New Taipei City 237, Taiwan; cjf6699@gmail.com (J.-F.C.); cyh0817@tmu.edu.tw (C.-Y.H.); 2 Division of Nephrology, Department of Internal Medicine, Taipei Medical University-Shuang Ho Hospital, New Taipei City 235, Taiwan; 3 Department of Nursing, Yuanpei University of Medical Technology, Hsinchu 300, Taiwan; 4 Renal Care Joint Foundation, New Taipei City 220, Taiwan; 5 Department of Internal Medicine, Armfulcare Hospital, Taoyuan City 320, Taiwan; 6 Division of Nephrology, Department of Medicine, Min-Sheng General Hospital, Taoyuan City 330, Taiwan; corey926@gmail.com; 7 Division of Rheumatology, Department of Internal Medicine, En Chu Kong Hospital, New Taipei City 237, Taiwan; eckdr.kuo@gmail.com; 8 Division of Pathology, Department of Internal Medicine, En Chu Kong Hospital, New Taipei City 237, Taiwan; 01393@km.eck.org.twtm; 9 Department of Biotechnology and Pharmaceutical Technology, Yuanpei University of Medical Technology, Hsinchu 300, Taiwan; hsucp@mail.ypu.edu.tw; 10 Department of Medical Laboratory Science and Biotechnology, Yuanpei University of Medical Technology, Hsinchu 300, Taiwan; 11 Department of Orthopaedic Surgery, National Taiwan University Hospital, Taipei 100, Taiwan; dtorth76@yahoo.com.tw; 12 Department of Orthopaedic Surgery, En Chu Kong Hospital, New Taipei City 237, Taiwan; dtorth65@yahoo.com.tw; 13 Division of Nephrology, Department of Medicine, Taipei Tzu Chi Hospital, Buddhist Tzu Chi Medical Foundation, New Taipei City 231, Taiwan; kuochenglu@gmail.com; 14 Graduate Institution of Biomedical and Pharmaceutical Science, College of Medicine, Fu Jen Catholic University, New Taipei City 242, Taiwan; 081551@mail.fju.edu.tw; 15 School of Medicine, Fu Jen Catholic University, New Taipei City 24205, Taiwan; skin@mail.fju.edu.tw; 16 Division of Cardiac Electrophysiology, Department of Cardiovascular Center, Cathay General Hospital, Taipei 106, Taiwan

**Keywords:** Caspase-1, COX2, cPLA2, IL-1β, inflammasome, NLRP3, p-cresyl sulfate, pyroptosis, reactive oxygen species, uremic vascular calcification

## Abstract

We formerly proved that uremic vascular calcification (UVC) correlates tightly with oxidative elastic lamina (EL) injury and two cell fates (apoptosis and osteocytic conversion) in smooth muscle cells (SMC) of chronic kidney disease (CKD) patients and eliminating p-cresyl sulfate (PCS)-activated intracellular ROS ameliorates the MAPK signaling pathway in a human arterial SMC (HASMC) model. Nonetheless, whether ROS scavenger attenuates PCS-triggered inflammasome activation and eicosanoid inflammation in the UVC process remains unknown. Patients with lower extremity amputation were categorized into CKD and normal control group according to renal function. We used immunohistochemistry stain to analyze UVC in arterial specimens, including oxidative injury (8-hydroxy-2′-deoxyguanosine (8-OHdG) and internal EL disruption), cytosolic phospholipase A2 (cPLA2), cyclooxygenase 2 (COX2), interleukin-1 beta (IL-1β), caspase-1 and NLRP3. To simulate the patho-mechanism of human UVC, the therapeutic effects of ROS scavenger on PCS-triggered inflammatory pathways was explored in a HASMC model. We found CKD patients had higher circulating levels of PCS and an increase in medial arterial calcification than the control group. In CKD arteries, the severity of UVC corresponded with expressions of oxidative EL disruption and 8-OHdG. Furthermore, coupling expressions of cPLA2 and COX2 were accentuated in CKD arteries, indicative of eicosanoid inflammation. Notably, tissue expressions of IL-1β, caspase-1 and NLRP3 were enhanced in parallel with UVC severity, indicative of inflammasome activation. From bedside to bench, ROS scavenger attenuates PCS-activated expressions of cPLA2/COX2, pro-caspase-1 and NLRP3 in the HASMC model. UVC as an inevitable outcome is predictive of death in CKD patients. Nonetheless, UVC remain pharmacoresistant despite the evolution of treatment for mineral-parathyroid hormone-vitamin D axis. Beyond the mineral dysregulation, the stimulation of pro-oxidant PCS alone results in eicosanoid inflammation and inflammasome activation. Concerning the key role of Caspase-1 in pyroptosis, cell fates of HASMC in uremic milieu are not limited to apoptosis and osteogenesis. In view of this, reducing ROS and PCS may act as a therapeutic strategy for UVC-related cardiovascular events in CKD patients.

## 1. Introduction

Mineral bone disorder (MBD) is a tricky systemic syndrome in chronic kidney disease- (CKD) patients, characterized by bone turnover disturbance, vitamin D deficiency, abnormal metabolism of calcium and phosphate, high circulating levels of intact parathyroid hormone (iPTH), and extra-osseous calcification in the vessel wall and soft tissue [1]. As time goes by, such uremic vascular calcification (UVC)-related cardiovascular (CV) events remain as the top leading causes of death in CKD patients, despite the evolution of treatment for mineral-PTH hormone-vitamin D axis [2]. To investigate the pathomechanism of UVC further, we have previously proved that UVC is correlated with renal function decline, retention of protein-bound uremic toxins (PBUTs), oxidative elastic lamina (EL) injury, contractile arterial smooth muscle cell (SMC) death and intracellular reactive oxygen species (ROS)-induced DNA damage of SMC [3]. Without the integrity of intima and EL, the direct exposure to circulating PBUTs determines the cell fate of SMC: apoptosis and osteogenic differentiation, culminating in irremediable cell death [3]. When it comes to the pro-oxidant effect of PTUTs, indoxyl sulfate and p-cresyl sulfate (PCS) are the two well-researched PBUTs, conferring CV and renal toxicity, that are deemed as long overlooked culprits in cardiorenal syndrome [4]. Meanwhile, we formerly proved the effect of PCS could drive osteogenesis via provoking ROS, pERK/pP38/pJNK MAPK pathways and NF-κB translocation to activate expressions of Runx2 and alkaline phosphatase in a human arterial SMC (HASMC) model [5]. Furthermore, PCS enhances alveolar cell death with tissue expressions of eicosanoid signaling molecules and ROS in our mouse model of uremic lung injury [6]. Notably, scavenging intracellular ROS attenuates above PCS-activated inflammatory signaling cascades in both HASMCs and alveolar cells [5,6]. Intriguingly, another uremic retention toxin trimethylamine N-oxide (TMAO) has been shown to act as a key player in cardiorenal syndrome. Emerging evidence indicates TMAO accelerates the activation of the nucleotide-binding oligomerization domain leucine-rich repeat proteins 3 (NLRP3) inflammasome in not only atherosclerosis but also UVC [7,8]. Additionally, NLRP2 was involved in regulating pro-inflammatory, pro-fibrotic and anti-apoptotic responses in renal tubular epithelial cells [9]. Despite previous documented evidence, whether ROS scavenger attenuates PCS-triggered eicosanoid inflammation and inflammasome cascade in UVC pathogenesis remains mysterious, leading to a lack of therapeutic targets. Accordingly, our primary purpose was to prove tissue expressions of cPLA2/COX2 and inflammasomes are activated in the UVC process using human models of vascular rings. From bedside to bench, the effect of intracellular ROS scavenging treatment for HASMC osteogenesis was investigated in PCS-triggered inflammatory signaling transduction.

## 2. Results

### 2.1. CKD Patients Had an Increase in Circulating Levels of PCS and Medial Arterial Calcification

The arterial specimens of 30 patients following amputation were included in the research. Demographic features and bio-clinical data were compared between CKD and normal control groups (Table 1). Underlying background characteristics were generally similar between the two study groups, apart from CKD-MBD-related manifestations. Our data demonstrated elevated plasma concentrations of PCS, calcium-phosphate product, non-hepatic alkaline phosphatase, and phosphate, showing significant differences between CKD patients and the normal controls. With regard to UVC, medial arterial calcification area in the CKD group was indeed higher than the control group. Elevated circulating levels of calcium-phosphate product reflect the diffuse precipitation of pro-calcifying particles in tissues, culminating in IVC-related systemic organ dysfunction. The correlation analysis of PCS, IS and the clinical parameters of UVC is summarized in Table 2. PCS and IS were significantly correlated with blood urea nitrogen, creatinine, estimated glomerular filtration rate, calcium-phosphate product, and medial arterial calcification area. Our data revealed that the heavy burden of PCS and pro-calcific milieu were associated with the severity of UVC.

### 2.2. CKD Arteries Exhibited More Serious Elastic Lamina (EL) Disruption in Parallel with UVC Severity

**Figure 1:**
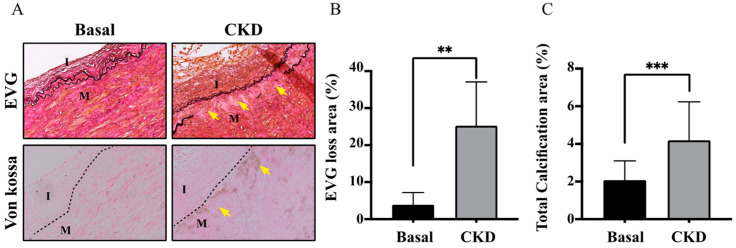
CKD arteries exhibited enhanced elastic lamina (EL) breach in parallel with UVC severity. We categorized arteries into CKD and basal groups. (**A**) The EVG stain was used to localize internal EL defect areas. The von Kossa stain was used to detect calcium deposits. Stretching and fragmentation of internal ELs with breaches in CKD arteries were adjacent to UVC regions and propagated along the media of the arterial wall. Compared with normal controls, yellow arrows indicated an incremental area loss internal EL in the CKD group, corresponding to the internal EL breach. (**B**,**C**) For the human experiments, we obtained 30 samples: controls (*n* = 12); CKD (*n* = 18). Control group, eGFR ≥ 60 mL/min; CKD, eGFR < 60 mL/min. Three random high-resolution fields were obtained for image analyses in each specimen. The Von Kossa and EVG staining were performed for quantitative analyses via ImageJ software. Data are presented as mean ± SD. The Student’s *t*-test for two groups is used to compare whether two samples are different. ** *p* < 0.01; *** *p* < 0.001. I, intima; M, media; CKD, chronic kidney disease; eGFR, EL, elastic lamina; EVG, elastic tissue fibers-Verhoeff’s Van Gieson; estimated glomerular filtration rate; UVC, uremic vascular calcification.

The elastic fibers and internal EL provide a boundary layer to separate endothelium, tunica intima (TI) from tunica media (TM) in the arteries. The internal EL consists of a lining and fine network of connective tissues bound together in a membrane, pierced with many openings that form a fenestrated sheet. Additionally, the internal EL accounts for not only cellular communications but also material transports across the TI. Therefore, the internal EL is responsible for the final defense line against pro-oxidant uremic toxins and pro-calcific particles in the blood of CKD patients. Based on the prior scenario of oxidative stress in CKD [6,10], we hypothesized that EL injury results in a dramatic influx of circulating PBUTs and pro-calcific particles into the arterial medial layer TM. As expected, CKD arteries had an incremental area of the von Kossa staining for medial arterial calcification area and decreased area of EVG staining, indicative of internal EL loss (Figure 1). As shown in Figure 1A, arteries of normal controls manifested histologically intact, whereas CKD arteries appeared to have advanced calcium deposition and were brown, black in color (Figure 1). Notably, the propagation of internal EL disruption could be found along the media of the arterial wall of CKD arteries. In addition, such CKD-related medial arterial calcification corresponded with the internal EL breaches (The yellow arrows in Figure 1A). We found that stretching and fragmentation of internal ELs with breaches were adjacent to medial arterial calcification lesions. Through the above internal EL breaches, circulating mineral particles leaked into the TM layer and contributed to the subsequent deposition of ectopic calcification. Current results show CKD arteries manifest more serious internal EL breaches and thereby catastrophic minerals leak into the TM to accelerate medial arterial calcification. The uremic milieu seems to erode the internal EL of the CKD arteries, resulting in a leaky intima, to accelerate systemic irremediable UVC. Why were CKD arteries prone to EL damage? Further experiments were required to prove circulating uremic toxins exert a pro-inflammatory and pro-oxidant effect on CKD arteries.

### 2.3. Concordant with UVC Severity, CKD Arteries Manifested Enhanced Expressions of COX-2/cPLA-2 and 8-OHdG, Indicative of Eicosanoid Inflammation and Oxidative Injury

**Figure 2:**
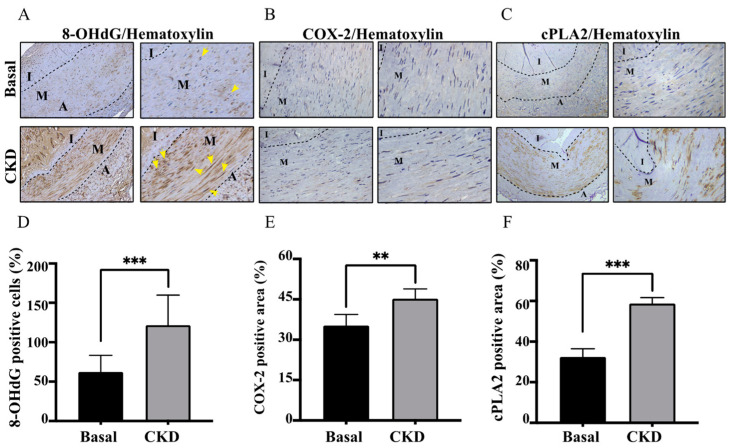
Effects of uremic vascular calcification on expressions of COX-2/cPLA-2 and 8-OHdG wthin arteries of CKD and controls. We categorized arteries into CKD and basal groups. ImageJ software was performed for immunohistochemical staining with quantitative analysis: (**A**) 8-OHdG (**B**) COX-2 and (**C**) cPLA-2, corresponding to bar graphs (**D**–**F**). The yellow arrows indicate 8-OHdG-positive cells. Thirty samples were collected for the human experiments: controls (*n* = 12); CKD (*n* = 18). Control group, eGFR ≥ 60 mL/min; CKD, eGFR < 60 mL/min. Three random high-resolution fields were obtained for image analyses in each specimen. Data are expressed as mean ± SD. The Student’s *t*-test for two groups is used to compare whether two samples are different. To compare more than three groups, Dunnett’s multiple comparisons test and ANOVA were applied to determine statistical significance. ** *p* < 0.01; *** *p* < 0.001. 8-OHdG, 8-hydroxy-2-deoxyguanosine; A, adventitia; I, intima; M, media; cPLA2, cytosolic phospholipase A2; eGFR, estimated glomerular filtration rate; CKD, chronic kidney disease; COX-2, cyclooxygenase-2.

As shown in Figure 2A,B,D,E, CKD-dependent UVC exerted an increase in coupling expressions of COX-2/cPLA-2, indicative of the activation of eicosanoid inflammation. Intriguingly, CKD arteries manifested an increase in UVC-dependent oxidative DNA damages of HASMCs through expressions of 8-OHdG (Figure 2C,F). The areas of oxidative HASMC DNA damages were concordant with UVC regions. Once direct exposure to pro-oxidant PBUTs, ROS provoke tissue damages by DNA modifications to inhibit repair functions and initiate maladaptive phenotypic transition in HASMCs [11,12]. Collectively, the above study results suggest that PBUT-induced ROS may serve as a key player in sending messages, leading to vascular oxidative injury and eicosanoid inflammation. Until now, whether other inflammatory pathways (e.g., NLRP3 inflammasome and Caspase-1 pathways) participated in vascular injury in human CKD arteries have been elusive.

### 2.4. Higher Productions of Caspase-1, IL-1β and NLRP3 Inflammasome in CKD-UVC Arteries and PCS-Stimulated HASMC

**Figure 3:**
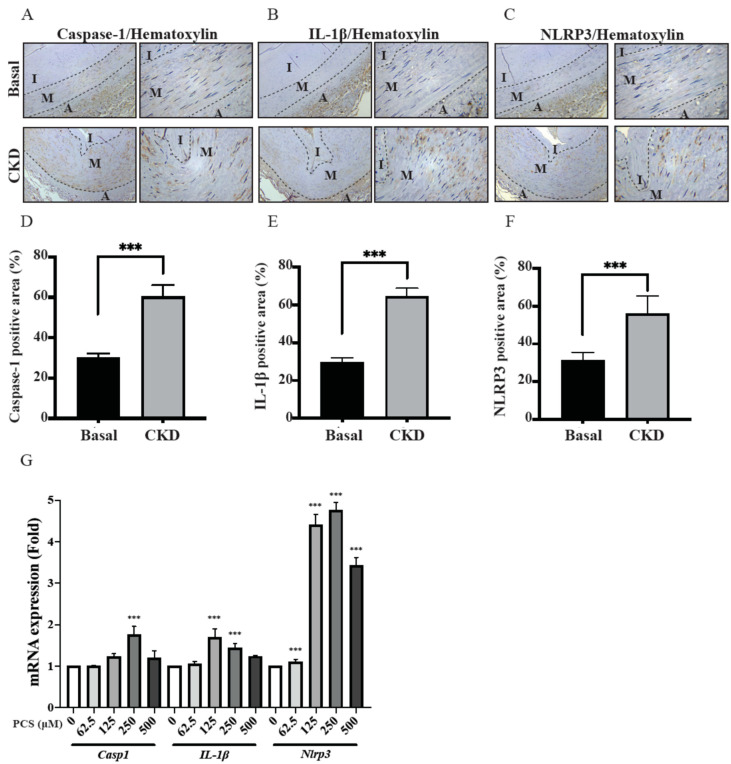
Expressions of caspase-1, IL-1β and NLRP3 inflammasome in CKD-UVC arteries and PCS-stimulated HASMC. We categorized arteries into CKD and basal groups. ImageJ software was performed for immunohistochemical staining with quantitative analysis: (**A**) caspase-1, (**B**) IL-1β and (**C**) NLRP3 inflammasome, corresponding to bar graphs (**D**–**F**). Thirty samples were used for human experiments: controls (*n* = 12); CKD (*n* = 18). Control group, eGFR ≥ 60 mL/min; CKD, eGFR < 60 mL/min. Three random high-resolution fields were obtained for image analyses in each specimen. (**G**) mRNA expressions of caspase-1, IL-1β and NLRP3 at the indicated concentrations of PCS stimulation in HASMC model. Data are expressed as mean ± SD. The Student’s *t*-test for two groups is used to compare whether two samples are different. To compare more than three groups, ANOVA with Dunnett’s multiple comparisons test was used to determine statistical significance. *** *p* < 0.001. PCS, p-cresyl sulfate; CKD, chronic kidney disease; eGFR, NLRP-3, NOD-, LRR- and pyrin domain-containing protein 3; estimated glomerular filtration rate; HASMC, human arterial smooth muscle cell; A, adventitia; I, intima; M, media.

In basic research, the uremic toxin TMAO is tightly correlated with NLRP3 activation in atherosclerosis and UVC [7,8]. However, there is scarce knowledge in the participation of PCS-activated inflammasome pathways in human CKD arteries. To outreach the prior findings to translational research, our data proved CKD-dependent UVC corresponds with higher productions of Caspase-1, IL-1β and NLRP3 in tissues (Figure 3A–F). NLRP3 inflammasome acts as a key regulator to stimulate caspase-1, which promotes IL-1β maturation and downstream pyroptosis, a form of cell death. Although current data provide human pathobiological evidence that the activation of caspase-1, IL-1β and NLRP3 inflammasome correspond to CKD-dependent UVC, whether circulating PBUTs trigger the above pathways remains unclear. To investigate it further, our experimental results in the HASMC model demonstrated that mRNA expressions of Caspase-1, IL-1β and NLRP3 were triggered by PCS in a concentration-dependent manner.

### 2.5. ROS Scavenger Attenuates PCS-Triggered Coupling Expressions of COX-2/cPLA-2 in HASMC Model

**Figure 4:**
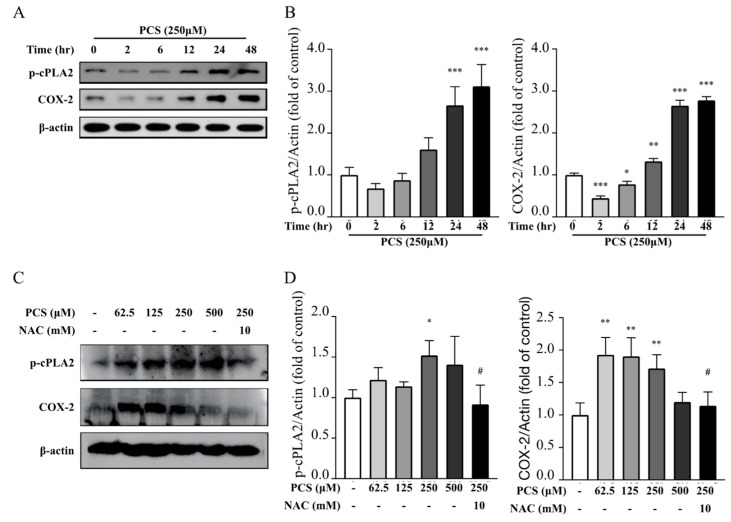
Influence of intracellular ROS scavenger on coupling expressions of COX-2/cPLA-2 induced PCS in HASMC model. (**A**,**C**) Different concentrations of PCS (0, 62.5, 125, 250, and 500 μM) were used to stimulate HASMCs for 24 h with or without ROS scavenger NAC treatment (10 mM, 30 min). After cellular disruption, HASMCs were analyzed using Western blotting with specific antibodies against COX-2 and cPLA-2. Intensities of bands were quantified using chemiluminescence detection reagent and densitometric analysis. (**B**,**D**) After quantification analysis, COX-2 and cPLA-2 were presented in fold change via bar graphs. Each experiment was replicated for three times. Data were expressed as mean ± standard deviation. To compare more than three groups, we used ANOVA and Dunnett’s multiple comparisons test to determine statistical significance. * *p* < 0.05, ** *p* < 0.01 and *** *p* < 0.001 indicated significant differences from the control group (0 μM PCS; 0.1% DMSO). # *p* < 0.05 indicated significant differences from the treatment group (250 μM). HASMC, human arterial smooth muscle cell; NAC—N-acetyl-L-cysteine; COX-2, cyclooxygenase-2; cPLA2, cytosolic phospholipase A2; PCS, p-cresyl sulfate.

Plenteous toxins exist abundantly in the uremic milieu that exert pro-oxidant effects on alveolo-capillary injury and renal fibrosis in tubulointerstitium through provoking ROS to trigger downstream COX-2/cPLA-2 inflammatory pathways, and ROS scavenging therapy may mitigate the above organ damages [6,10]. To explore the influence of the intracellular ROS scavenger on eicosanoid inflammation triggered by PCS, we treated HASMC cultures with PCS at different concentrations of PCS (0, 62.5, 125, 250, and 500 μM) for 24 h, with or without the treatment of the non-specific ROS scavenger NAC (10 mM). Western blotting data showed that PCS accentuates the coupling expressions of COX-2/cPLA-2 in HASMCs (Figure 4). Notably, NAC suppressed PCS-stimulated COX-2/cPLA-2 activation in HASMCs. While facing PCS exposure, ROS may serve as key players in signaling transduction that triggers downstream inflammatory cascades to accentuate UVC.

### 2.6. ROS Scavenger Attenuates PCS-Triggered Expressions of Pro-Caspase-1 and NLRP3 in HASMC Model

**Figure 5:**
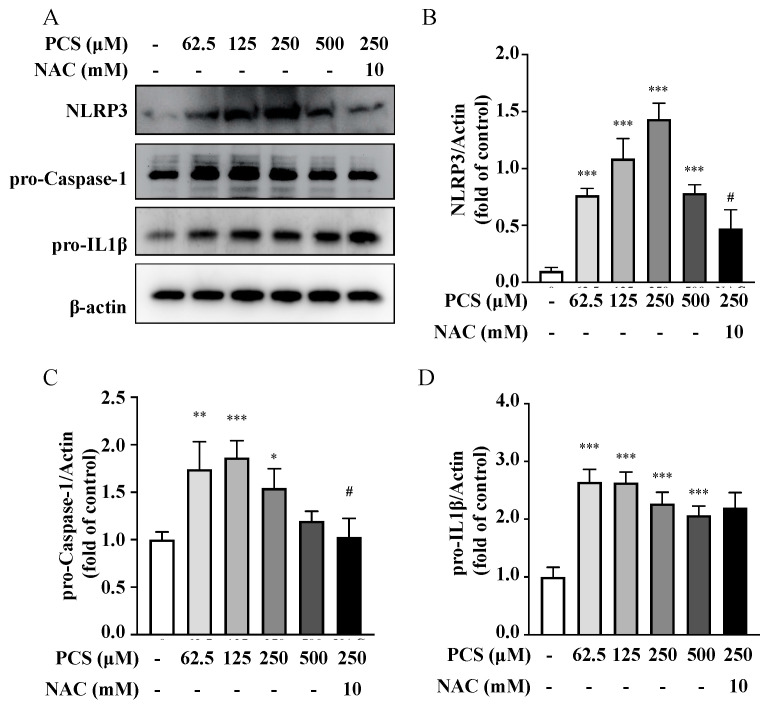
The influence of intracellular ROS scavenger on NLRP3 inflammasome, pro-caspase-1, and pro-IL 1β stimulated by PCS in HASMC model. (**A**) Various concentrations of PCS (0, 62.5, 125, 250, and 500 μM for 24 h) with or without NAC treatment (10 mM, 30 min) were performed in HASMC cultures. After cellular disruption, HASMCs were analyzed using Western blotting with specific antibodies against NLRP3 inflammasome, pro-caspase-1, and pro-IL 1β. Intensities of bands were quantified using by chemiluminescence detection reagent and densitometric analysis. (**B**–**D**) After quantification analysis, NLRP3 inflammasome, pro-caspase-1, and pro-IL 1β were presented in fold change via bar graphs. Each experiment was replicated for three times. Data were expressed as mean ± standard deviation. To compare more than three groups, ANOVA with Dunnett’s multiple comparisons test was used to determine statistical significance. * *p* < 0.05, ** *p* < 0.01 and *** *p* < 0.001 indicated significant differences from the control group (0 μM PCS; 0.1% DMSO). # *p* < 0.05 indicated significant differences from the treatment group (250 μM). HASMC, human arterial smooth muscle cell; NAC—N-acetyl-L-cysteine; NLRP-3, NLR family pyrin domain containing 3; PCS, p-cresyl sulfate.

In our prior HASMC model, intracellular ROS induced by PCS did trigger osteogenic conversion, and ROS scavenger inhibited PCS-activated osteoblast-specific protein alkaline phosphatase and osteogenic transcription factor runx2 [5]. To explore the influence of the intracellular ROS scavenger on the production of inflammasome induced by PCS, we treated HASMCs cultures with PCS at various concentrations of PCS (0, 62.5, 125, 250, and 500 μM) for 24 h with or without ROS scavenger NAC treatment (10 mM, 30 min). Western blotting data showed that PCS activated the intracellular expression of NLRP3 inflammasome, pro-caspase-1 and pro-IL 1β in HASMCs (Figure 5). Notably, our findings illustrated PCS aggravated expressions of intracellular pro-caspase-1 and NLRP3 (Figure 5B,C). Intriguingly, NAC could not inhibit PCS-induced activation of pro-IL 1β in HASMCs. Thus, the stimuli of pro-IL 1β may not be limited to the pro-oxidant effect of PCS. Collectively, the above results demonstrate PCS-induced ROS serves as key player to activate inflammasome pathways and inflammatory cascade in HASMCs, culminating in irremediable UVC.

## 3. Discussion

CKD-related uremic milieu accelerates the progression of extra-osseous vascular calcification and bone fragility, yet UVC serves as a key player between cardiorenal events and CKD-MBD [13,14,15]. Medial UVC contributes to CV stiffening, intractable hypertension, coronary heart diseases, diastolic heart failure and premature CV death [16]. Conventional medical treatment, such as statins and anti-hypertensives, fail to effectively treat dialysis patients with UVC, and therefore CV diseases remain the leading cause of mortality [17,18,19,20]. Likewise, standard treatments based on mineral-PTH hormone-vitamin D axis seem ineffective for outcome improvement in UVC patients [20]. Therefore, the necessity for the development of treatments that focus on UVC patho- mechanisms in clinical practice remains urgent. Why do patients with CKD tend to suffer from UVC? Growing evidence points out that UVC is principally induced by HASMC while facing active inducers, including minerals, uremic toxins, pro-oxidants, and diverse pro-inflammatory mediators [3]. Beyond the concentration gradient-dependent paracellular passive transport, the internal EL as the innermost layer of intima affects molecular transport between TI and TM [21]. To protect the arterial wall integrity from the circulating PBUT influx, the dense elastic fiber layer EL acts as the defense line for the TM layer. Oxidative EL disruption allows a catastrophic PBUT leak to drive intricate inflammatory responses in HASMCs, leading to systemic irremediable UVC. To acquire an in-depth understanding and novel insights into the pathobiology and mechanism of UVC, we used not only human medium-sized muscular arteries but also a HASMC model to test the therapeutic effects of intracellular ROS scavenger on eicosanoid pathways and inflammasome activation. In this cardiorenal translational research, issues of great import deserve further discussion.

### 3.1. Pro-Oxidant PCS-Induced Internal EL Disruption as an Emerging Key Player of UVC Progression

Vascular oxidative injury is reminiscent of an imbalance between the overproduction of ROS and insufficient antioxidant abilities [22]. Uremic toxins play a pivotal role in ROS generation in the scenario of CKD [23]. Such ROS starts tissue damages through driving modifications for both proteins and DNA, resulting in abnormal EL structures and functions, impairing HASMC repair ability, and a vicious UVC circle [3,5,22,23,24]. In the current study, we demonstrate UVC corresponds with the disruption of internal EL and 8-OHdG, indicative of oxidative injury. Once internal EL breaches become more serious, breakpoints allow catastrophic leaks of PBUTs and mineral particles into TM to induce HASMC inflammation, death, medial arterial calcification and overwhelming systemic UVC. If above documented hypothesis is true, how could we prevent the arterial wall from the dramatic influx of circulating PCS and minerals? In the past decades, CKD-MBD therapies focused mainly on mineral-PTH hormone-vitamin D axis, such that UVC-associated mortality remains at the top of leading causes in CKD population. Hung et al. reported that CKD patients with secondary hyperparathyroidism increased expression of sclerostin that inhibits Wnt 10b/Wnt 16 signaling pathway, leading to activating osteoclastic bone resorption (tartrate-resistant acid phosphatase isoform 5b) and inactivating osteoblastic bone formation (procollagen type I propeptides), bone inflammation and low bone density, and ultimately adverse clinical events [25]. In light of this, PCS-driven UVC remains intractable. Toward this end, HASMCs were treated with PCS in this study. Indeed, ROS scavenger effectively attenuates PCS-upregulated expressions of COX-2/PLA-2, caspase-1 and NLRP3 inflammasome.

### 3.2. Higher Circulating Levels of PCS Is in Parallel with UVC Severity, Oxidative EL Injury, Coupling Expressions of COX-2/cPLA-2, and ROS Scavenger Inhibits PCS-Induced Eicosanoid Inflammation

Under the protection of intact EL integrity, HASMCs in the normal vascular TM are completely separated from uremic toxins and mineral particles. The scenario of CKD-MBD consists of plenteous uremic toxins that exert pro-inflammatory and pro-oxidant abilities. We formerly reported PBUTs cause alveolo-capillary injury and renal fibrosis in tubulointerstitium via producing ROS to initiate downstream eicosanoid inflammation signaling pathways, and antioxidant therapy may mitigate the above organ damages in our prior research [6,10]. Intriguingly, we found the effect of PCS alone enhances intracellular ROS levels, transcription factor Runx2 and osteoblast-specific protein alkaline phosphatase in the HASMC model without the participation of pro-calcific particles (calcium, phosphate, and precipitation of calcium phosphate) [3]. Accordingly, we utilized previous studies for the present cardiorenal research. Compared with normal controls, CKD patients had indeed higher circulating levels of PCS and medial arterial calcification area, indicative of UVC severity (both *p*-values < 0.01) (Table 1). After that, CKD arteries had higher expressions of oxidative damages (8-OHdG and EL disruption) and COX-2/cPLA-2, indicative of the eicosanoid inflammation (all *p*-values < 0.01) (Figure 1 and Figure 2). Furthermore, ROS scavenger inhibits PCS- induced coupling expressions of COX-2/cPLA-2 in HASMC model (Figure 4). Collectively, we prove PCS-induced ROS may account for key players in UVC-related eicosanoid inflammation.

### 3.3. CKD-UVC Arteries Exert Higher Expressions of Caspase-1, IL-1β and NLRP3 Inflammasome, and ROS Scavenger Inhibits PCS-Activated Caspase-1 and NLRP3 Inflammasome

**Figure 6:**
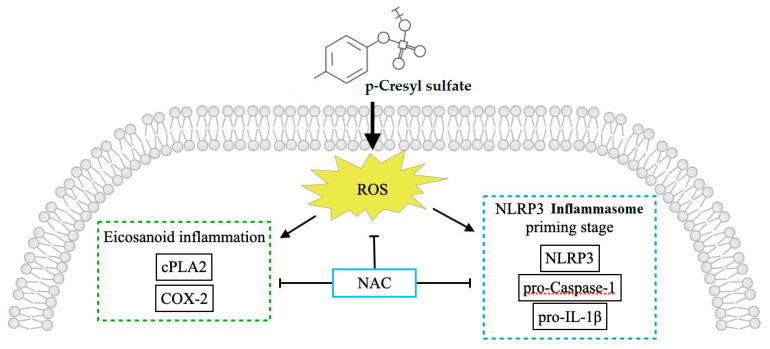
New insights into the contribution of uremic compounds in cardiorenal syndrome: PCS-triggered ROS as potential therapeutic targets in HASMC. The direct exposure to circulating uremic toxin PCS triggers intracellular ROS that activates eicosanoid inflammation and inflammasome pathways in HASMC. Notably, intracellular ROS scavenger NAC attenuates expressions of cPLA2/COX-2, NLRP-3 and pro-Caspase 1. Thus ROS may act as the key player in the inflammatory message transduction in response to PCS. In viewing of this, PCS and intracellular ROS may be potential therapeutic targets for UVC in patients with CKD. cPLA2, cytosolic phospholipase A2; PCS, p-cresyl sulfate; COX-2, cyclooxygenase-2; HASMC, human arterial smooth muscle cells; NAC, N-acetyl-L-cysteine; NLRP-3, NOD-, LRR- and pyrin domain-containing protein 3; CKD, chronic kidney disease; ROS, reactive oxygen species.

In the process of cellular damages, a class of cytosolic protein complexes termed as inflammasomes formed to mediate host immunity [26,27]. Proteolytic cleavage of dormant procaspase-1 into active caspase-1 occurs during inflammasome activation, which induces pyroptosis, a form of cell death [27]. Additionally, the cytokine precursor pro-IL-1β is converted by active caspase-1 into mature and biologically active IL-1β. Being a critical pro-inflammatory mediator, mature IL-1β participates in diverse immune reactions, including the recruitment of innate immune cells to the site of inflammation and modulation of adaptive immune cells. With regard to the critical role in above inflammatory cascades, NLRP3 inflammasome has been known to act as a therapeutic target for vascular insults [28]. Gene expressions of uremic toxins can be enhanced by NLR-inflammasome-activated caspase-1 and other pro-inflammatory cytokines [1]. Meanwhile, gene expressions of uremic toxins are significantly upregulated in CKD and CV diseases [29]. Glorieux et al. indicate free PCS shows the highest association with CV outcomes in CKD patients [30]. In view of above vicious cycle between inflammasome cascade and uremic toxins in patients with cardiorenal syndrome, there should be UVC-activated inflammasome pathways in CKD. Toward this end, we demonstrate that human CKD arteries indeed exert higher expressions of caspase-1, IL-1β and NLRP3 inflammasome, corresponding with the UVC severity and EL disruption (Figure 1 and Figure 3). After that, our HASMC data reveal eradicating PCS-triggered intracellular ROS results in decreased expressions of intracellular pro-caspase-1 and NLRP3 (Figure 5). Taken together, this is a brand new pathobiological study to prove PCS-induced ROS serve as key messenger molecules to activate inflammasome pathways and eicosanoid inflammation in HASMCs, providing new insights into the contribution of uremic compounds in cardiorenal syndrome (Figure 6).

## 4. Materials and Methods

### 4.1. Patients and Arterial Specimens

**Figure 7:**
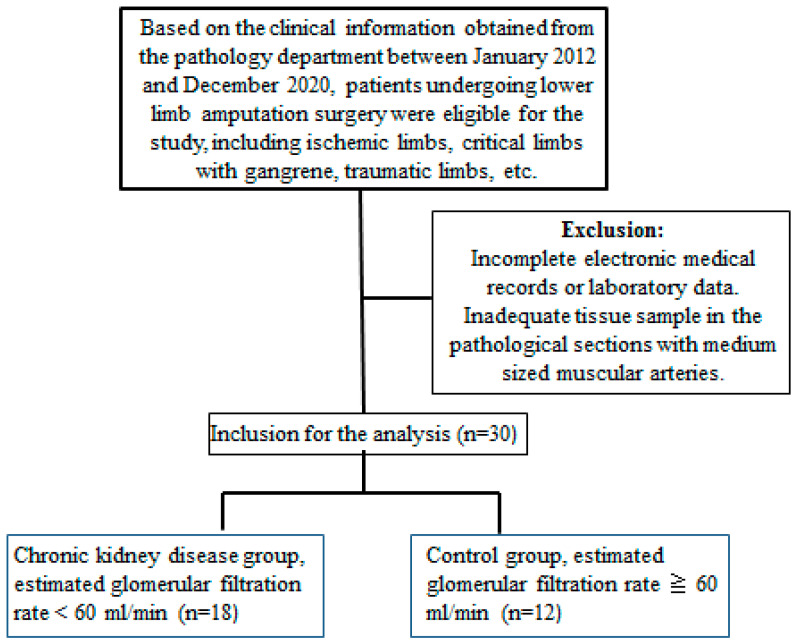
Flowchart of the study.

The arterial specimens obtained from patients following lower limb amputation surgery were enrolled in this study, including those with acute ischemic limbs, critical limb with gangrene, traumatic limbs, etc. We recorded bio-clinical and laboratory parameters for further investigation: age, gender, systolic blood pressure, diastolic blood pressure, blood urea nitrogen, creatinine, estimated glomerular filtration rate (eGFR), alanine aminotransferase, glucose, potassium, calcium, phosphate, alkaline phosphatase. Because the timing of the blood draw test was very individualized, we excluded patients with missing data. After a careful chart review in our study, patients with hepatobiliary diseases were excluded and thereby raised circulating alkaline phosphatase levels were determined to be non-hepatic origins, such as bone events, UVC or CKD-MBD [31]. Non- hepatic alkaline phosphatase is a byproduct derived from osteoblast activation. An increase in hydrolysis of mineralization inhibitor pyrophosphate by alkaline phosphatase enhances vascular calcification [32]. Circulating pro-calcific particles include calcium, phosphate, and precipitation of calcium phosphate. We classified study patients into the following two groups by eGFR for further comparison: CKD (eGFR ≤ 60 mL/min) and normal controls (eGFR > 60 mL/min). The eGFR was estimated by an equation developed by the CKD Epidemiology Collaboration. The enrollment flowchart was as follows (Figure 7):

### 4.2. Reagents for Immunohistochemistry (IHC) Staining and Cell Models

We analyzed pathological sections of medium-sized muscular arteries from patients who underwent amputation surgery. A detailed evaluation for histopathological characterizations was performed for all amputation specimens. The hematoxylin and eosin stain was used to assess tissue morphology. We used the paraffinize blocks, de-paraffinized in xylene and rehydrated in a graded alcohol series to create 3 μm tissue sections. We captured the images with Nikon Digital Camera Microscope (Nikon, Tokyo, Japan). The histological sections of human artery vessel rings were stained following the Kit User Guides: (1) Von Kossa stain for calcification (CVK-1-IFU, ScyTek Laboratories, Logan, UT, USA); (2) elastic tissue fibers-Verhoeff’s Van Gieson (EVG) stain for elastic tissue fibers (1:200 dilution; Ab150667, Abcam, Cambridge, UK); (3) COX-2 antibody for eicosanoid inflammation (1:200 dilution; Sc-1745, Santa Cruz, CA, USA); (4) cPLA-2 antibody for eicosanoid inflammation (1:200 dilution; sc-454, Santa Cruz, CA, USA); (5) 8-hydroxy-2′-deoxyguanosine (8-OHdG) stain for oxidative DNA damage (1:200 dilution; Bs-1278R, Bioss Antibodies, Woburn, MA, USA) (6) Caspase-1 antibody for inflammasome activation (1:200 dilution; 06-503-I, Milipore, Temecula, CA, USA); (7) NLRP3 (1:200 dilution; AG-20B-0014, AdipoGen, San Diego, CA, USA) for inflammasome activation; and (8) IL-1 β (1:200 dilution; AB-401-NA, R&D, Minneapolis, MN, USA), for inflammasome activation. The procedure of performing IHC staining was designed according to the manufacturer’s protocol (BioTnA, Kaohsiung, Taiwan). The blocking step is necessary before primary antibodies (TA00C2, BioTnA, Kaohsiung, Taiwan). We stained tissue sections with primary antibodies, followed by HRP- conjugated anti-rabbit secondary antibody (TAHC02D, BioTnA, Kaohsiung, Taiwan). We detected the expression levels using the TAlink mouse/rabbit polymer detection system (TAHC04D, BioTnA, Kaohsiung, Taiwan). We converted all glass slides of tissue sections to digital virtual slides through the slide scanner (Motic Easyscan Digital Slide Scanner, Hong Kong, China) with high precision autofocus. Within the arterial medial layer (M), the Von Kossa positive regions (brown color) were divided by total areas (Figure 1). The medial arterial calcification area was expressed as a percentage (%). The Von Kossa and EVG staining procedure was performed with the manufacturer’s instructions. We deparaffinized sections and hydrate sections with distilled water for Von Kossa staining. Silver Nitrate Solution (5%) was used to incubate slides for 30–60 min while exposing to either ultraviolet light or incandescent light at 75 watts or greater. The light source within 2 feet (61 cm) of the slide was maintained to ensure the best results during the Silver Nitrate staining procedure. Slides were then rinsed in three changes of distilled water and incubated in Sodium Thiosulfate Solution (5%) for 2 min. We stained tissue sections with Nuclear Fast Red Solution for 5 min after rinsing for 2 min in running tap water, followed by two changes of distilled water. Next, slides were rinsed for 2 min in running tap water followed by two changes of distilled water. Finally, we mounted slides with synthetic resin after quick dehydration in the absolute alcohol. Slides were placed in working Elastic Stain Solution for 15 min after deparaffinization for EVG staining. Afterwards, we rinsed slides in running tap water until no excess stain remained on the slide. Slides were checked microscopically for proper differentiation and rinsed in running tap water after the step of dip in differentiating solution 15–20 times and rinsed in tap water. Next, we mounted slides in Sodium Thiosulfate Solution for 1 min and rinsed in running tap water. Slides were then stained with Van Gieson’s Solution for 2–5 min and rinsed in two changes of 95% alcohol. Finally, we mounted slides with synthetic resin after quick dehydration in the absolute alcohol. Elastic fibers were stained blue to black through standard operating. The disrupted area of elastic fibers was quantified with the following equation: EVG negative area (yellow arrow) was divided by EVG positive area (blue- black wavy lines), expressed as a percentage (Figure 1). The area percentage of IHC stain was quantified with the ImageJ version 1.48 v image analysis software (National Institutes of Health, MD, USA). We collected thirty human samples for the experiments: Basal (*n* = 12) and CKD (*n* = 18). Three random high-resolution fields were obtained in each sample for image analyses.

### 4.3. Cell and Treatments

The primary HASMCs were purchased from the American Type Culture Collection (ATCC PCS-100-012, Rockville, MD, USA). HASMCs were cultured in the vascular cell basal medium (ATCC PCS-100-030) with a vascular smooth muscle cell growth kit (ATCC PCS-100-042) at 37 °C in a 5% CO_2_ incubator. The HASMC passage number in the experiments was P7–P9. In the treatment group, HASMCs were pretreated with NAC prior to PCS administration. NAC was always present during PCS stimulation.

### 4.4. RNA Isolation and Quantitative Real-Time PCR

According to the manufacturer’s instruction, we extracted total RNA using Trizol Reagent (Ambion, Austin, TX, USA) and cDNAs with the Superscript Vilo kit (Invitrogen, Waltham, MA, USA). The reverse transcription reaction was performed at 42 °C for 30 min, at 99 °C for 5 min, and then cooled to 4 °C. We held the PCR mixture at 94 °C for 2 min and then cycled 30 times at 94 °C for 30 s, 55 °C for 30 s, and 72 °C for 2 min, followed by 10 min at 72 °C at the final cycle. We performed real-time PCR assays with TaqMan Universal PCR Master Mix and gene expression assays from Applied Biosystems (Waltham, MA, USA). We normalized gene expression with human gapdh as the endogenous control. We used the 2Δct method to analyze data and expressed data as arbitrary units or as fold difference.

### 4.5. Measurement of Circulating PCS Levels

We purchased NAC from Sigma-Aldrich (St. Louis, MO, USA) and PCS from Alsachim (Illkirch-Graffenstaden, France), respectively. We pretreated blood samples (50 μL) with 1400 μL acetonitrile (ACN) to precipitate proteins, followed by centrifugation at 13,400× *g* for 20 min at 4 °C. We evaporated each tube of the supernatant via a spin vacuum instrument. The above lyophilized samples were re-dissolved in 200 μL 30% ACN aqueous solution with 0.1% formic acid (FA). We analyzed PCS concentrations with a tandem mass spectrometry system (Thermo Finnigan TSQ Quantum tra Mass Spectrometer, Thermo Fisher Scientific Inc., Hampton, NH, USA). We equipped the tandem mass spectrometry system with a Micro ESI ion source, which was set at 3.0 kV, coupled with an Accela 1250 UHPLC analytical system (Thermo Fisher Scientific Inc.). PCS mixtures in the above samples were sequentially injected into the UHPLC via the Accela 1250 autosampler and separated using a Shiseido HPLC CAPCELL PAK C18 MGII column (150 mm × 1.5 mm, 3.0 μm, Tokyo, Japan). As described in our prior research, multiple reaction monitoring was used as scanning mode for quantification [5].

### 4.6. Statistics

Continuous variables and categorical variables were expressed as mean ± standard deviation, and number (%), respectively. We used the ImageJ version 1.48 v image analysis software (National Institutes of Health, Bethesda, MD, USA) to quantify the area percentage of IHC images. We used the Xcalibur software (version 2.2, Thermo-Finnigan Inc., San Jose, CA, USA) to acquire the MS spectra and control the mass spectrometer. Data were analyzed with the GraphPad Prism 7 (GraphPad Software, Inc., San Diego, CA, USA) or SPSS version 22.0 (IBM, Armonk, NY, USA). The Student’s *t*-test for two groups is used to compare whether two samples are different in terms of a quantitative variable. To compare more than three groups, statistical significance was determined by ANOVA with Dunnett’s multiple comparisons test. For each of the experiments, the statistical significance was determined by *p* values less than 0.05.

## 5. Conclusions

UVC, an inevitable complication in CKD patients, remains a therapeutic dilemma. Despite the evolution of treatment for mineral-parathyroid hormone-vitamin D axis, UVC remain pharmacoresistant in CKD patients. The direct exposure to circulating uremic toxins triggers vascular oxidative stress, leading to EL breaches and subsequent inflammatory cascades in HASMCs. Beyond the mineral dysregulation, the stimulation of pro-oxidant PCS alone results in eicosanoid inflammation and inflammasome activation. With regard to the key role of Caspase-1 in pyroptosis, cell fates of HASMC in uremic milieu are not limited to apoptosis and osteogenesis. In view of this, ROS and PCS may serve as potential therapeutic targets for UVC-related CV events in CKD patients.

## 6. Patients

In accordance with bio-clinical data from the electronic medical charts between January 2016 and December 2020, patients following lower limb amputation surgery were enrolled in this study including acute ischemic limbs, critical limb with gangrene, traumatic limbs, etc. For further investigation, amputation specimens of lower-extremity arteries were excluded for patients without complete information. Then, we classified patients into the following two groups using eGFR for further investigation: CKD (eGFR ≤ 60 mL/min) and normal controls (eGFR > 60 mL/min).

## Figures and Tables

**Table 1:** Comparisons of underlying bio-clinical manifestations between CKD and control groups ^a^.

Variables	Controls (*n* = 12)	CKD (*n* = 18)	*p*-Value
Age (years)	67.3 ± 15.4	65.9 ± 12.8	0.78
Male, *n* (%)	9 (75.0)	13 (72.2)	0.87
Diabetes, *n* (%)	4 (33.3)	14 (77.8)	<0.05
Cardiovascular disease, *n* (%)	4 (33.3)	7 (38.9)	0.77
Heart failure, *n* (%)	3 (25.0)	5 (27.8)	0.87
Anemia, *n* (%)	5 (41.7)	10 (55.6)	0.48
Systolic blood pressure (mmHg)	130.8 ± 34.0	143.1 ± 25.7	0.27
Diastolic blood pressure (mmHg)	75.4 ± 16.1	82.9 ± 19.7	0.43
Blood urea nitrogen (mg/dL)	18.0 ± 6.1	47.2 ± 23.8	<0.01
Creatinine (mg/dL)	0.9 ± 0.2	3.9 ± 2.4	<0.01
eGFR (mL/min) ^b^	80.9 ± 13.8	22.6 ± 15.5	<0.01
Sodium (mmol/L)	135.1 ± 4.2	136.4 ± 5.4	0.46
Potassium (mmol/L)	4.2 ± 0.7	3.9 ± 0.5	0.22
Glucose (mg/dL)	151.6 ± 43.9	243.9 ± 113.2	<0.05
Alanine aminotransferase (IU/L)	29.2 ± 21.0	18.5 ± 11.7	0.10
Alkaline phosphatase (IU/L)	125.6 ± 35.4	176.4 ± 60.9	<0.05
Calcium (mg/dL)	9.0 ± 0.4	8.8 ± 0.9	0.62
Phosphate (mg/dL)	2.9 ± 0.4	5.0 ± 1.2	<0.01
Calcium-phosphate product ^c^	26.1 ± 2.8	43.4 ± 9.7	<0.01
p-cresyl sulfate	1.2 ± 0.9	15.2 ± 8.1	<0.01
Medial arterial calcification area (%) ^d^	2.1 ± 1.0	4.2 ± 1.9	<0.01

Categorical variables are represented by number (%). Continuous variables were presented as mean ± SD. The Student’s *t*-test is used for study groups to compare whether two samples are different. ^a^ CKD, chronic kidney disease; CKD group, eGFR less than 60 mL/min; Control group, eGFR more than 60 mL/min. ^b^ eGFR, estimated glomerular filtration rate. ^c^ Calcium-phosphate product = calcium × phosphate. ^d^ Medial arterial calcification area was quantified by the equation: the von Kossa positive region was divided by the arterial medial layer area (percentage).

**Table 2:** The correlation analysis between clinical parameters of uremic vascular calcification, indoxyl sulfate and p-Cresyl sulfate.

	p-Cresyl Sulfate (ug/mL)	Indoxyl Sulfate (ug/mL)	*p*-Value
Creatinine (mg/dL)	0.50	0.52	<0.01
eGFR (ml/min)	0.71	0.67	<0.01
Blood urea nitrogen (mg/dL)	0.42	0.41	<0.05
Calcium-phosphate product	0.76	0.70	<0.01
Medial arterial calcification area (%)	0.42	0.41	<0.05

eGFR = estimated glomerular filtration rate. The eGFR is estimated by an equation developed by the Chronic Kidney Disease Epidemiology Collaboration. Medial arterial calcification area was quantified by the equation: the von Kossa positive area was divided by the area of arterial medial layer (percentage). Spearman test was used for the correlation analysis.

## Data Availability

The datasets for this article are not publicly available because they contain information about patent application in Taiwan (utility model patent 110203393 and in-vention patent 110111409). Requests to access the datasets should be directed to Jia-Feng Chang, cjf6699@gmail.com.
